# Identifying latent subgroups of primary head injury: an explorative latent class analysis on neuropathologically examined medico-legal autopsy cases

**DOI:** 10.1007/s12024-024-00913-5

**Published:** 2024-11-14

**Authors:** Essi Laakko, Petteri Oura

**Affiliations:** 1 Department of Forensic Medicine, University of Helsinki, P.O. Box 21, Helsinki, FI-00014 Finland; 2 Forensic Medicine Unit, Finnish Institute for Health and Welfare, P.O. Box 30, Helsinki, FI-00271 Finland

**Keywords:** Forensic pathology, Traumatic brain injury, Central nervous system, Post mortem

## Abstract

Traumatic brain injury (TBI) is a significant global health concern and frequently encountered in medico-legal autopsies. Previous studies suggest that certain TBI subtypes are more likely to co-occur than others. Therefore, we aimed to explore the potential of latent class analysis (LCA) to identify and characterize primary head injury combinations in neuropathologically examined medico-legal autopsy cases. The dataset comprised 78 cases from the Forensic Medicine Unit of the Finnish Institute for Health and Welfare over the period of 2016–2022. Data on background and circumstantial characteristics as well as primary and secondary head and brain injuries were collected from police documents, medical records, general autopsy reports and neuropathology reports. Latent class solutions with two to five classes were explored to identify clustering of primary head injuries among the sample. The dataset comprised 69.2% males and the median age was 49 years. In LCA, the solutions appeared reasonable, and each class appeared to represent a distinct TBI profile. The two-class solution was found to fit the present dataset best. Class 1 was characterized by older age, presence of an underlying CNS disease, and less diverse primary head injuries; these were interpreted as suggestive of lower traumatic forces. Class 2 was characterized by male sex and assaults as a prominent injury circumstance; subarachnoid and intracerebral/ventricular haemorrhages and contusions were classified exclusively into this class. In conclusion, this study identified two distinct subgroups of primary head injuries. Understanding typical injury combinations related to distinct circumstances could assist not only forensic pathologists but also clinicians treating TBI patients. However, the present latent class solution should not be interpreted as “ground truth”, but instead further research is needed.

## Introduction

Traumatic brain injury (TBI) represents a major global health issue, significantly contributing to the disability and mortality associated with traumatic injuries [1]. TBI encompasses a diverse range of pathologies arising from various mechanisms [2]. These injuries can be classified not only as focal or diffuse based on the extent of brain damage [3] but also as contact injuries or inertial (acceleration-deceleration) injuries [4]. Primary injuries are the immediate result of a traumatic force (e.g. fractures, intracranial haemorrhages), while secondary injuries are delayed or indirect consequences of the initial trauma (e.g. oedema, raised intracranial pressure) [2]. Frequent causes of TBI include falls, assaults and traffic accidents [5]. Depending on the type of injury, the highest risk groups are children, young men and older adults [6].

The circumstances of TBI and the characteristics of patients vary, resulting in diversity in injury combinations. For example, road traffic fatalities have been associated with an abundant, simultaneous occurrence of fractures, subdural haemorrhage (SDH) and subarachnoid haemorrhage (SAH) [7]. As typical acceleration-deceleration injuries, SDH and diffuse traumatic axonal injury (dTAI) often occur in similar circumstances, although SDH has been reported to occur more likely in simple falls and assaults, whereas dTAI is more typical in falls from a considerable height and traffic accidents [4]. It therefore appears that certain TBI subtypes are more likely to co-occur than others.

Knowledge of common injury combinations related to specific circumstances and patient groups could facilitate the work of forensic pathologists as well as clinicians. In the medico-legal setting, further knowledge on typical head injury combinations could expedite the cause-of death investigation and potential judicial proceedings in TBI cases.

Of statistical methods that aim to classify cases into subgroups (i.e. classes or clusters), latent class analysis (LCA) is widely used for identifying distinct groups within a heterogenous population [8]. Even though cluster analysis has previously been applied in certain medico-legal research themes (e.g. child homicide [9], urban firearm injuries [10], and sudden maternal death [11]), applications in forensic neuropathology and TBI deaths have been scarce. It would be essential to be able to determine which head injuries are frequently associated with one another and elucidate the circumstances underlying these injury combinations.

The purpose of this study was to explore the potential of LCA to identify and characterize primary head injury combinations among neuropathologically examined medico-legal autopsy cases. The objectives were to describe the application of LCA in a medico-legal TBI dataset, and to report the best-fitting latent class solution in the present dataset. We expected to identify several subgroups that differ in background variables and head injuries.

## Materials and methods

### Material

In Finland, sudden and unexpected deaths, as well as deaths due to a suspected accident, homicide, suicide, medical or surgical adverse event, occupational disease, or war, are reported to the police for a medico-legal cause-of-death investigation. Medico-legal autopsies are ordered by the police and performed by forensic pathologists in the Forensic Medicine Unit of the Finnish Institute for Health and Welfare. Of all deaths in Finland, approximately 15% undergo a medico-legal autopsy [12, 13].

The material of this study comprised cases that were referred to a medico-legal autopsy in the Helsinki office of the Forensic Medicine Unit (metropolitan office with over 3000 autopsies annually [14]) over the period 2016—2022 and whose autopsy was associated with a full neuropathological examination of the formalin-fixed brain by a board-certified neuropathologist. Cases with an acute head injury (i.e. scalp haemorrhage, fracture of the cranium/facial bones/cervical spine, traumatic intracranial haemorrhage, contusion, or traumatic axonal injury (TAI)) observed either in the general autopsy or neuropathology were included in the analysis.

Approval for the study was obtained from the Finnish Institute for Health and Welfare (THL/1802/6.02.00/2023). The data were collected by accessing the case files in an electronic information system that includes all data of the medico-legal autopsy cases.

### Background and circumstantial characteristics

Sex (male/female) and age at death (years) were obtained from the cause-of-death investigation report submitted by the police. Police documents and available medical history were queried for the presence of any underlying CNS disease (yes/no) and remote CNS injury (yes/no), as well as the general circumstance of the recent injury occurring prior to death (fall/assault/traffic accident/other/unknown/no document of recent injury). Medico-legal case files were used to obtain the height (cm), weight (kg) and fresh brain weight (g) of the case, all measured during the general autopsy, as well as to calculate postmortem interval (days). Finally, the manner of death was recorded from the death certificate (disease/accident/homicide/other).

### Head injuries

The presence of the following primary head injuries (defined as acute extracranial, cranial, or intracranial injuries) were obtained from the general autopsy report and neuropathology report: scalp haemorrhage, fracture of the cranium/facial bones/cervical spine, traumatic intracranial haemorrhage (epidural, subdural, subarachnoid, intracerebral/ventricular), contusion, and TAI (yes/no for each). β-amyloid precursor protein (β-APP) stain was performed in all cases, and the TAI variable included both focal and diffuse TAI. As for secondary injuries, the following variables were collected from the neuropathology report: brain oedema in either microscopic or macroscopic examination; hypoxic-ischaemic neuronal injury based on pycnotic, eosinophilic or necrotic neurons in haematoxylin-eosin stain; and vascular axonal injury in β-APP stain (yes/no for each). Primary and secondary injuries were recorded in a dichotomous format (yes/no), regardless of severity or extent; mild cases were also included.

### Statistical analysis

Our general aim was to explore and demonstrate the potential of LCA to identify primary head injury combinations among the sample. LCA is generally applied to statistically classify cases into distinct groups (i.e. classes or clusters) based on prespecified variables of interest [8, 15]. We performed LCA on the basis of primary head injury variables; other variables were not included as the aim was to reveal potential primary injury combinations irrespective of background and circumstantial data. R studio version 4.4.0 for Windows [16] and the poLCA package were used. We explored models with two to five classes, documenting the fit statistics of each model. Cases were classified in the most probable class according to the highest posterior class membership probability given by the model. The following parameters were obtained from the poLCA output to evaluate model fit: Bayesian information criterion (BIC), Akaike information criterion (AIC), likelihood ratio/deviance statistic (G^2^), log-likelihood, Chi-square goodness of fit (X^2^). We also recorded relative class sizes and average posterior class membership probabilities in order to monitor the robustness of the models. The solution that was considered best fit in the present dataset [15] was chosen as the final model. However, due to the explorative nature of the study, LCA models with two to five classes were all presented to facilitate comparisons.

The distributions of categorical variables were presented as percentages and frequencies, and those of continuous variables as medians and interquartile ranges (IQRs). As the dataset was relatively small, statistical differences between the classes were analysed by means of conservative and non-parametric tests (i.e. Fisher’s Exact Test, Fisher-Freeman-Halton Exact Test, and Mann-Whitney U test, as appropriate). P values < 0.05 were considered statistically significant. SPSS Statistics version 27 (IBM, Armonk, NY, USA) were used to perform the analyses except for the LCA. Excel version 2405 (Microsoft, Redmond, WA, USA) was used to create bar charts illustrating the prevalences of primary head injuries in the classes.

## Results

The full dataset comprised 78 medico-legal autopsy cases that had suffered a head injury prior to death and were referred to a neuropathological examination. The median age of the cases was 49 years (IQR 27–73) and 69.2% were male (Table 1). The median postmortem interval was 6 days (IQR 3–9) and the neuropathological examination comprised a median of 17 brain regions sampled (IQR 15–18). Regarding medical history, 41.0% of the cases had a previous disease of the CNS and 28.2% had a remote injury to the CNS. In 60.3% of the cases, the autopsy referral or police documents specified a recent injury event, most common circumstances being assault (26.9%) and fall (20.5%). The most common primary head injuries were scalp haemorrhage (78.2%), SAH (39.7%), TAI (26.9%), fracture (25.6%), and SDH (10.3%).

**Table 1:** Characteristics of the full sample

Characteristic	All (*n* = 78)
Male sex	69.2 (54)
Age (y)	49 (27–73)
Postmortem interval (d)	6 (3–9)
Body height (cm)	172 (159–177)
Body weight (kg)	70 (55–82)
Brain weight (g)	1404 (1283–1523)
Medical history	
Previous disease of the CNS	41.0 (32)
Remote injury to the CNS	28.2 (22)
Circumstance of recent injury	
Fall	20.5 (16)
Assault	26.9 (21)
Traffic accident	5.1 (4)
Other/unknown injury	7.7 (6)
No recent injury specified	39.7 (31)
Manner of death	
Disease	44.9 (35)
Accident	23.1 (18)
Homicide	19.2 (15)
Other	12.8 (10)
Primary head injuries	
Scalp haemorrhage	78.2 (61)
Fracture (cranial, facial, or cervical)	25.6 (20)
Epidural haemorrhage	1.3 (1)
Subdural haemorrhage	10.3 (8)
Subarachnoid haemorrhage	39.7 (31)
Intracerebral/ventricular haemorrhage	11.5 (9)
Contusion	7.7 (6)
Traumatic axonal injury	26.9 (21)
Secondary brain injuries	
Brain oedema	42.3 (33)
Vascular axonal injury	20.5 (16)
Hypoxic-ischaemic neuronal injury	76.9 (60)

Numbers are percentages with frequencies, or medians with interquartile ranges

CNS = Central nervous system

**Fig. 1:**
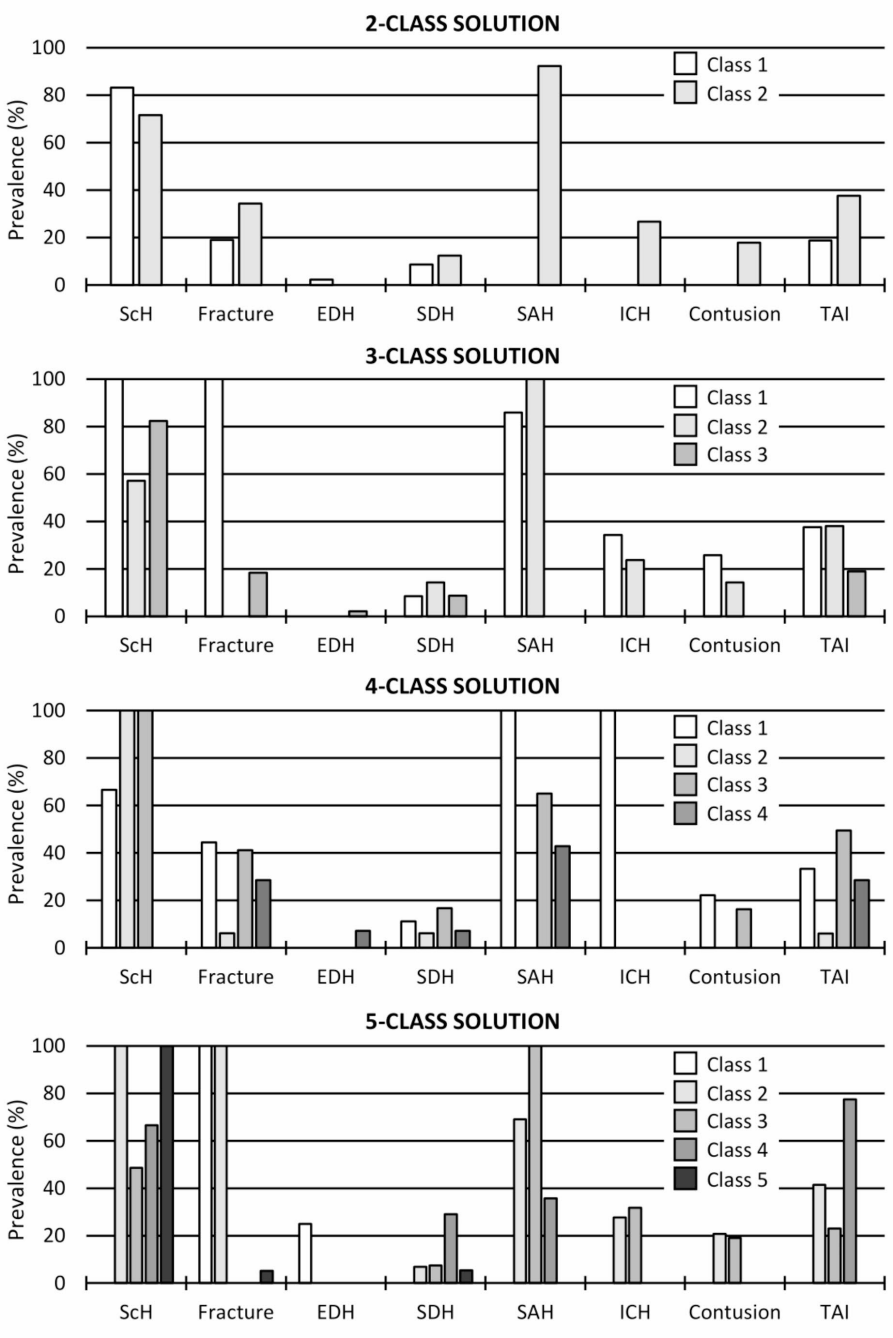
Distribution of primary injuries in the latent class models with two to five classes. The two-class solution was found to fit the present data best. Fracture comprises cranial, facial, and cervical fracture. ICH comprises intraparenchymal and intraventricular haemorrhage. EDH, Epidural haemorrhage; ICH, Intracerebral haemorrhage; SAH, Subarachnoid haemorrhage; ScH, Scalp haemorrhage; SDH, Subdural haemorrhage; TAI, Traumatic axonal injury

LCA was applied to explore models with two to five classes. The distributions of primary injuries in the explored models are presented in Fig. 1. In brief, the two-class solution appeared to have one class in which primary injuries were less diverse (Class 1) and another class with a wider range of intracranial injuries (Class 2). The three-class solution had one class with less diverse injuries (Class 3) and two classes characterized by a higher diversity of intracranial injuries (Classes 1 and 2). The four- and five-class solutions were able to fraction out classes with more nuanced primary injury combinations.

Fit statistics of the explored latent class models are presented in Table 2. Based on fit statistics, the model with two classes presented the lowest AIC and BIC values, as well as the highest likelihood ratio and Chi-square goodness of fit. Relative class sizes were acceptable (≥ 41% of the sample) and average posterior probabilities were high (≥ 0.97). As such, the two-class solution was taken forward as the statistically best model for the present dataset.

**Table 2:** Fit statistics of the latent class models

Number of classes	AIC	BIC	G^2^	X^2^	Log-likelihood	Class sizes	Average posterior probabilities
**2**	**531.5**	**571.6**	**75.7**	**147.1**	**-248.7**	**0.41/0.59**	**1.00/0.97**
3	541.3	602.6	67.6	122.1	-244.7	0.29/0.59/0.12	1.00/0.97/1.00
4	542.9	625.4	51.1	66.0	-236.4	0.32/0.18/0.37/0.13	0.93/0.86/0.94/1.00
5	562.6	666.3	52.9	80.2	-237.3	0.06/0.08/0.23/0.22/0.41	1.00/0.98/0.93/0.95/1.00

AIC = Akaike information criterion, BIC = Bayesian information criterion, G^2^ = Likelihood ratio/deviance statistic, X^2^ = Chi-square goodness of fit

Bold indicates the best solution

Among the subgroups identified in the two-class model, Class 1 consisted of 46 cases and Class 2 comprised 32 cases. The characteristics of the classes are presented in Table 3. There were 65.2% males in Class 1 and 75.0% in Class 2. There was a 10-year difference in median age between the classes, though it was not statistically significant (59 vs. 49 years, *p* = 0.192). Upon reviewing medical history, a significantly higher prevalence of underlying CNS disease was observed in Class 1 (52.2% vs. 25.0%, *p* = 0.020).

**Table 3:** Comparison of background characteristics and primary and secondary head and brain injuries between the classes identified in the best latent class solution

Characteristic	Class 1 (*n* = 46)	Class 2 (*n* = 32)	*P* value
Male sex	65.2 (30)	75.0 (24)	0.457
Age (y)	59 (31–75)	49 (19–57)	0.192
Postmortem interval (d)	7 (5–9)	5 (2–8)	0.158
Body height (cm)	171 (156–178)	172 (160–177)	1.000
Body weight (kg)	74 (55–82)	64 (54–84)	0.651
Brain weight (g)	1371 (1276–1526)	1437 (1301–1524)	0.694
Medical history			
Previous disease of the CNS	52.2 (24)	25.0 (8)	0.020
Remote injury to the CNS	23.9 (11)	34.4 (11)	0.322
Circumstance of recent injury			
Fall	26.1 (12)	12.5 (4)	
Assault	19.6 (9)	37.5 (12)	
Traffic accident	2.2 (1)	9.4 (3)	
Other/unknown injury	6.5 (3)	9.4 (3)	
No recent injury specified	45.7 (21)	31.3 (10)	0.130
Manner of death			
Disease	54.3 (25)	31.3 (10)	
Accident	21.7 (10)	25.0 (8)	
Homicide	13.0 (6)	28.1 (9)	
Other	10.9 (5)	15.6 (5)	0.172
Primary head injuries			
Scalp haemorrhage	82.6 (38)	71.9 (23)	0.279
Fracture (cranial, facial, or cervical)	19.6 (9)	34.4 (11)	0.189
Epidural haemorrhage	2.2 (1)	0.0 (0)	1.000
Subdural haemorrhage	8.7 (4)	12.5 (4)	0.710
Subarachnoid haemorrhage	0.0 (0)	96.9 (31)	< 0.001
Intracerebral/ventricular haemorrhage	0.0 (0)	28.1 (9)	< 0.001
Contusion	0.0 (0)	18.8 (6)	0.004
Traumatic axonal injury	19.6 (9)	37.5 (12)	0.119
Secondary brain injuries			
Brain oedema	34.8 (16)	53.1 (17)	0.162
Vascular axonal injury	17.4 (8)	25.0 (8)	0.570
Hypoxic-ischaemic neuronal injury	82.6 (38)	68.8 (22)	0.179

Numbers are percentages with frequencies, or medians with interquartile ranges. CNS = Central nervous system

In Class 2, an assault scenario was the most common circumstance of the recent injury (37.5%; Table 3). Accident and homicide were common manners of death. As for primary head injuries, cases in Class 2 had a higher prevalence of fractures, SDH, SAH, and intracerebral/ventricular haemorrhage, contusion, and TAI (Fig. 1). Of these, the differences in SAH (96.9% vs. 0.0%, *p* < 0.001), intracerebral/ventricular haemorrhage (28.1% vs. 0.0% *p* < 0.001) and contusion (18.8% vs. 0.0%, *p* = 0.004) were statistically significant.

In contrast, cases in Class 1 were characterized by falls (26.1%; Table 3) and lack of a specified recent injury in autopsy referrals and police documents (45.7%). Disease was the commonest manner of death. The most typical primary head injury was scalp haemorrhage; others were less common and included fractures, EDH, SDH and TAI (Fig. 1).

## Discussion

A total of 78 neuropathologically examined medico-legal autopsy cases, all of whom had suffered a head injury prior to death, were involved in this study. The objectives were to describe the application of LCA in a medico-legal TBI dataset, and to report the best-fitting latent class solution in the present dataset. Latent class solutions with two to five classes were explored. The complexity of the models increased along with the number potential of classes. In this relatively small dataset, the two-class solution showed the best fit statistics and was selected as the final solution. The two classes of the final model showed distinct characteristics and statistically significant differences in the prevalences of underlying CNS disease, SAH, intracerebral/ventricular haemorrhage and contusion. As such, our analysis gives grounds for several remarks.

This study is among the first to apply LCA in a forensic neuropathology and medico-legal TBI context. As certain TBI subtypes are more likely to co-occur than others (e.g. fractures, SDH and SAH in road traffic fatalities [7], or SDH and dTAI in acceleration-deceleration scenarios [4]), further data on typical brain injury combinations could expedite cause-of death investigations and potential judicial proceedings in TBI cases. These aspects provide us with a theoretical justification for the explorative application of LCA in the forensic neuropathology context.

In our dataset, the latent class solutions appeared reasonable from the practical point of view; each class appeared to represent a distinct TBI profile. As is often the case, the models became more complex as the number of classes was increased [15]. The two-class solution showed the best fit statistics and was selected as the final model. Of note is the fact that our dataset was relatively small and our primary injury variables were dichotomous; the present solution should not be interpreted as “ground truth”, but instead further research is needed. Multicenter efforts with detailed TBI variables (e.g. estimation of survival time by means of immunohistochemical stains [17]) may produce valuable datasets for future use. Future research should also aim to take into account the location, accumulation, severity and extent of head injuries. However, as the two-class solution was selected as the best for the present dataset, we introduce its classes and discuss the class differences below.

As for medical history, underlying CNS disease was more prevalent in Class 1, affecting 52.2% of the cases. The association between accidental deaths and CNS diseases, particularly Alzheimer’s and vascular dementia, has been shown previously [18]. Neurological and cognitive impairment significantly affect balance predisposing patients to falls [19]. Similarly, these impairments may affect driving ability, increasing the risk of traffic accidents. Previous research has identified older age combined with a CNS disease as a significant risk factor to falls [19], consistent with our finding of a 10-year higher median age in Class 1.

The circumstance of the recent head injury was often assault or traffic accident in Class 2, possibly indicating a greater force of trauma than in Class 1, in which falls and unspecified circumstances prevailed. We speculate that undocumented events mainly comprise fragile patients’ simple falls at home, which are not reported to the healthcare services and, consequently, do not appear in our records. However, our dataset did not differentiate between falls from different heights, which prevents us from determining whether the falls in Class 2 could have been more severe and have caused worse injuries. In general, we speculate that cases in Class 2 have encountered on average greater traumatic forces, leading to more diverse primary head injury combinations.

SAH was a prominent finding in Class 2, affecting 96.9% of the cases. Acceleration, deceleration and twisting movement are typical causes of SAH that may present in varying severity, intensity and distribution [2]. In previous studies, SAH has been the commonest injury in falls [20], assaults [21], homicides [22], and traffic accidents [23, 24]. However, no significant differences in the incidence of SAH between the injury circumstances have been found [20].

Consistent with the observations in this study, contusion and SAH are associated with each other [2]. Similar to SAH, contusions were seen in Class 2 only, affecting 18.8% of the cases. Contusion has been reported to be more prevalent in traffic accidents than falls [20], which corresponds to our findings indicating that contusions were only observed in Class 2, where traffic accidents were more prevalent. Moreover, traumatic intracerebral/ventricular haemorrhage was solely seen in Class 2, with a prevalence of 28.1%. Both intracerebral and intraventricular hemorrhages are associated with dTAI and mainly observed in severe head injuries [2]. This and previous research [25] align with our hypothesis on the potentially greater traumatic forces encountered by the cases of Class 2.

Knowledge on typical injury combinations related to specific circumstances and case profiles could facilitate the work of police and forensic pathologists estimating the course of events leading to a person’s unnatural death. For clinicians, knowledge of co-occurring injuries may provide the opportunity to make evidence-based predictions and treatment decisions, aiming to prevent disability and death.

A strength of the study were the comprehensive neuropathological examinations performed by a board-certified neuropathologist. The dataset comprised medical history, general circumstances of TBI, as well as primary and secondary head and brain injuries documented during the general autopsy and neuropathological examination, enabling comparisons between classes. Additionally, LCA provided an objective approach to the dataset; it has rarely been utilized as a statistical method in forensic neuropathology before. The main limitations were the relatively small sample size and lack of details on the severity and extent of head injuries. Unfortunately, specific details on injury circumstances were also limited; for example, falls from significant height and from standing position were grouped into the same category, and the detailed mechanisms of assaults were not available to us. Finally, we did not have data on injuries to other parts of the body than the head.

## Conclusion

This study explored the potential of LCA to identify and characterize primary head injury combinations in a dataset of 78 medico-legal autopsy cases. A two-class solution appeared to fit the present data best. Class 1 was characterized by older age, presence of an underlying CNS disease, and less diverse primary head injuries; these were suggestive of lower traumatic forces. Male sex, assaults and hypothetically greater traumatic forces were typical in Class 2; neuropathological examinations revealed subarachnoid and intracerebral/ventricular haemorrhages and contusions exclusively in this class. Understanding typical injury combinations related to distinct circumstances could assist not only forensic pathologists but also clinicians treating TBI patients. However, the present latent class solution should not be interpreted as “ground truth”, but instead further research is needed.

## Key points

1. Certain head and brain injuries are more likely to co-occur than others.
2. This study describes the application of latent class analysis in a traumatic brain injury dataset.
3. The analysis identified two distinct head injury groups in the dataset.
4. Knowledge of typical injury combinations could assist both forensic pathologists and clinicians.

## Data Availability

The dataset is not made public due to local privacy regulations.
